# Taking the perspectives of many people: Humanization matters

**DOI:** 10.3758/s13423-020-01850-4

**Published:** 2020-12-14

**Authors:** Tian Ye, Fumikazu Furumi, Daniel Catarino da Silva, Antonia Hamilton

**Affiliations:** 1 Institute of Cognitive Neuroscience, University College London, London, UK; 2 Japan Society for the Promotion of Science, and Graduate School of Human Development and Environment, Kobe University, Kobe, Japan

**Keywords:** Perspective-taking, In-group, Dehumanization, Social cognition, Motivation

## Abstract

In a busy space, people encounter many other people with different viewpoints, but classic studies of perspective-taking examine only one agent at a time. This paper explores the issue of selectivity in visual perspective-taking (VPT) when different people are available to interact with. We consider the hypothesis that humanization impacts on VPT in four studies using virtual reality methods. Experiments 1 and 2 use the director task to show that for more humanized agents (an in-group member or a virtual human agent), participants were more likely to use VPT to achieve lower error rate. Experiments 3 and 4 used a two-agent social mental rotation task to show that participants are faster and more accurate to recognize items which are oriented towards a more humanized agent (an in-group member or a naturally moving agent). All results support the claim that humanization alters the propensity to engage in VPT in rich social contexts.

**Supplementary Information:**

The online version contains supplementary material available at 10.3758/s13423-020-01850-4.

Walking into a busy shop, the shopper might encounter a number of other figures, such as a shop worker, a friend, and a shop mannequin, who all have different visual perspectives on the scene. This paper examines how people engage in visual perspective-taking when encountering multiple different agents with different social characteristics. In particular, we aim to examine the tension between claims that we automatically consider the visual perspective of people we encounter (Samson, Apperly, Braithwaite, Andrews, & Bodley Scott, 2010) and the suggestion that not all people we encounter are fully humanized (Haslam, 2006), together with the real-world observation that we often meet more than one person at a time.

Visual perspective-taking (VPT) is the process of determining whether another person can see an object and what the object looks like to that person (Flavell, 1977). Many cognitive studies over the past decade have suggested that at least some forms of VPT are automatic and occur without top-down control (Elekes, Varga, & Király, 2016, 2017; Freundlieb, Kovács, & Sebanz, 2016, 2018; Samson et al., 2010; Furlanetto, Becchio, Samson, & Apperly, 2015; Surtees, Apperly, & Samson, 2016; Surtees, Samson, & Apperly, 2016). Samson et al. (2010) found an ‘altercentric intrusion effect’ when participants were asked to report the number of discs on the walls and a human agent saw a different number of discs than they did. Recently, Ward and colleagues found that recognition of rotated letters was easier when the letters were oriented towards another person (Ward, Ganis, & Bach, 2019). Similarly, lexical decisions on rotated words are easier when the words are oriented to another person (Freundlieb et al., 2018; Freundlieb, Sebanz, & Kovács, 2017). Thus, in different contexts, the presence of another person can either interfere with (Samson’s task) or facilitate (social mental rotation task) participant’s judgments of what they themselves can see. These studies have typically used rapid reaction time measures in tightly controlled environments and suggest rapid or even automatic mechanism of processing other’s visual perspectives.

This contrasts with studies using tasks that give more time for thought in fewer trials. For example, researchers found that adults are more likely to draw an *E* on their own forehead to be readable by another person if the participant feels less powerful (Galinsky, Ku, & Wang, 2005) or if the confederate is from in-group (Vaes, Paladino, & Leyens, 2004). Similarly, young children use more metalizing words when describing in-group members (McLoughlin & Over, 2017), and adults are more likely to attribute secondary emotions to in-group members (Demoulin et al., 2009). Finally, people will spontaneously take the perspective of a human more than a robot in a single-trial online study (Zhao, Cusimano, & Malle, 2016). These results can be summarized in terms of humanization (Gray, Gray, & Wegner, 2007)—that is, the theory that people do not attribute as many human abilities, including emotion and perspective-taking, to robots and out-group members compared with in-group members. However, this has rarely been tested in cognitive VPT tasks.

Only a few recent studies hinted that varying the identity of the agent might impact perspective-taking behaviours. For instance, Savitsky and colleagues (2011) were interested in how social closeness would influence perspective-taking. They invited participants to a set of communication tasks with either a friend (or their spouse) or a stranger, where in each trial they needed to stand in the partner’s perspective to work out the true meaning of an ambiguous statement. Interestingly, the results showed no better performance when participants interacted with a close other compared with a stranger. Similarly, Todd, Hanko, Galinsky, and Mussweiler (2011) found that people sometimes performed worse in the in-group context when perspective taking is involved. They found that when contemplating a factual statement from a same-ethic group member, people made more errors than when interacting with a different-ethic group member. Similarly, guiding an in-group member out of a maze took much longer time (mean = 70.86 s) than that for an out-group member (mean = 55.73 s). On the other hand, Ferguson, Brunsdon, and Bradford (2018) recently found that adults encountered a higher altercentric intrusion effect from an adult agent than from a child agent in a dot-counting task (Samson et al., 2010). This result suggested that people may have a stronger propensity to engage in the perspective of a similar other. However, to date, no study has systematically tested how varying the humanization of a target can alter perspective taking in classic cognitive tasks.

This present paper aims to integrate these perspectives and understand how VPT works in more naturalistic contexts for different agents or more than one agent. The majority of previous studies has used a static photo of a neutral person as a stimulus, and gives us little information about how perspective-taking works in real-world contexts where there are many people who can move. We use virtual reality to test the hypothesis that humanization acts as a gateway to VPT and that participants selectively take the perspectives of agents who are more humanized, even in rapid response tasks.

Using virtual reality, we implemented two VPT tasks with moving human-like agents and less human-like agents (a robot or statue). This allows us to systematically test whether different levels of humanization change the propensity to engage in VPT. First, we adopted the widely used director task (Keysar, Barr, Balin, & Brauner, 2000; Keysar, Lin, & Barr, 2003; Wu & Keysar, 2007), which is sensitive to the performance differences in adults (Dumontheil, Küster, Apperly, & Blakemore, 2010) to measure spontaneous VPT for humans, robots, in-group, and out-group members. The director task tests spontaneous perspective-taking in a conversational context. The participants were usually presented with a set of open shelves, with some shelves blocked from view. Participants need to follow the instruction of a ‘director’ who stands behind the shelves, and must take or move an object from one of the shelves. To select the correct object, participants need to be aware that the director cannot see the objects in the blocked shelves, so that they need to focus on those objects in the common view. In our study, we manipulated the humanness level of the director. The aim of these experiments is to see whether different levels of ‘humanness’ could have an impact on VPT when people interact with one agent at a time.

In a second step, we adapted a new social mental rotation task (Ward et al., 2019) into a two-person VPT selection task, and pushed VPT further by testing how participants select which perspective to take when two agents have two competing perspectives. Based on Ward’s previous task, we created a virtual room with two agents sitting opposite each other by the table, and participants need to identify normal or mirror-reversed letters that have various orientations. We hypothesize that in this experimental setting, participants would be more likely to take the perspective of an agent with a higher level of ‘humanness’. Thus, when the letter is facing toward such an agent, participants should recognize it more accurately or faster.

## Experiment 1

Experiment 1 tests whether people have a stronger propensity to take the perspective of a virtual agent that looks human, compared with one that looks like a robot, using a version of the director task. In this task, participants are instructed by a director to move certain objects and must realize that some objects are hidden from the director, so as to avoid errors. The 2 × 2 factorial within-subjects design included the factors agent (human, robot) and condition (experimental trials, control trials). We collected error rate and reaction times, but consider error rate as the primary outcome measure because the task was not speeded.

### Method

#### Participants

Female participants (mean age = 21.7 years, *SD* = 2.50 years) were recruited from the Institute of Cognitive Neuroscience (ICN) participants database. They gave written informed consent before taking part and the study was approved by the ICN ethics committee (ICN-AH-PWB-3-3-14a). The sample size (*n* = 30) was predefined based on a power analysis showing this provides at least 80% power to detect a medium effect size (Cohen’s *f* = 0.25).

#### Materials

**Fig. 1:**
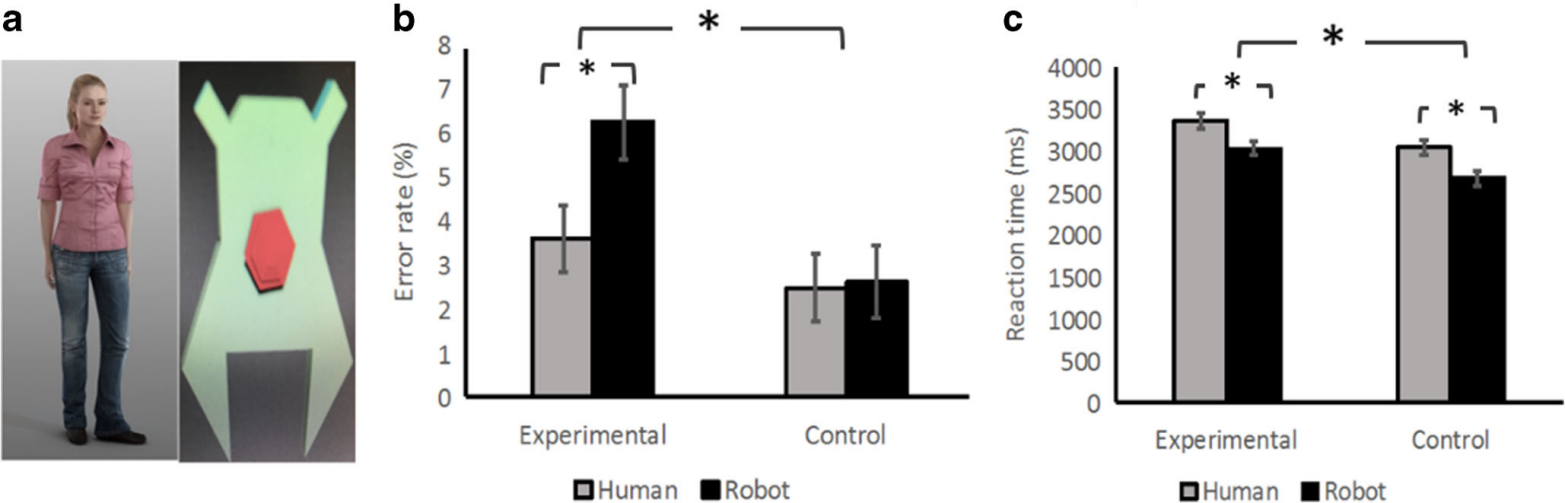
Experiment 1. **a** Human agent and robot agent. **b** Error rates for Experiment 1. **c** Reaction ties for Experiment 1. Error bars indicate the standard errors. * *p* < .05, ** *p* < .01

The experimental paradigm was created using Vizard 5 virtual reality software, running on a Windows 7 computer, with an Oculus DK2 Head-mounted display (HMD). The two virtual ‘directors’ are shown in Fig. 1a. The human agent looked like a European woman, and her voice was recorded by a native British speaker. The robot agent was a flat figure with one ‘eye’ and a voice from Robot Voice Generator (https://lingojam.com/RobotVoiceGenerator).

#### Procedure

Participants first gave their consent and had time to familiarize themselves with the HMD. As part of this familiarization, they could explore a VR space with a set of shelves. The items on the shelves were all visible from the front, but some were occluded from the back of the room. Participants were instructed to explore the whole room and count the visible objects, to allow them to understand that objects could be occluded from different viewpoints. Next, participants entered the VR ‘factory’, where a conveyor belt carried sets of shelves between two large machines with items (dice or balls) on the shelves, and no one could see the shelves before they emerged from the first machine (see Figure 1 in the Supplementary Info). Participants were told that two inspectors worked in the factory and would look at the items on the shelves to find faults, then tell the participant which items to remove. For one training trial, the experimenter explicitly asked the participant which objects could be seen from the director’s perspective.

On each trial, the shelves moved on the conveyor to the center of the screen while the director walked in, turned towards the conveyor, and inspected the objects. The human director made a short head-turn action while the robot made a mechanical sound to convey looking. The director asked the participant to take a target object (e.g., ‘Take the small ball’), and the participant moved a hand-shaped cursor to select and object, making it disappear. Then the shelves with the remaining objects then moved away while the director walked out. Each trial had a total duration of approx. 10 seconds.

We contrasted trials needing perspective-taking (experimental trials) and control trials. In the experimental trials, a distractor object was hidden from the director by an occluding panel; for example, if the director says ‘take the small die’, the participant must realize that the director cannot see the smallest die and must take the midsized die instead (see Figure 1 in the Supplementary Info). In contrast, for control trials no perspective-taking was needed as no distractor object is occluded. We combine these two trial types with the two directors giving a 2 × 2 factorial design. Each condition had 24 trials for a total of 96 trials, which were presented in fully random order. After the VR task was complete, participants filled in a short questionnaire which assessed their sense of social presence in VR (Usoh, Catena, Arman, & Slater, 2000) and their impressions of the virtual agent. See section S2 in the Supplementary Info. for a detailed description of the questions and the questionnaire results.

### Results

Error rates were analyzed using a 2 × 2 repeated-measures analysis of variance (ANOVA), with agent (human, robot) and condition (experimental, control) as within-subjects factors (see Fig. 1b). Holm corrections were used for post hoc multiple comparisons. Main effects and the interaction were all significant—agent: *F*(1, 29) = 10.54, *p* = .003, η_p_^2^ = .27; condition: *F*(1, 29) = 5.60, *p* = .03, η_p_^2^ = .16; interaction: *F*(1, 29) = 6.64, *p* = .02, η_p_^2^ = .19. The post hoc analysis shows that in the experimental trials, participants made more errors with the robot agent than with the human agent, *t*(29) = 4.34, *p* < .001, whereas there was no difference between agents in the control trials, *t*(29) = .23, *p* = .82. A bootstrap analysis was performed to take into account the possibility that the data may not be normally distributed and gave equivalent results; both main effects and the interaction were significant.

The mean reaction times (RTs) were calculated from correct responses after excluding extreme values (±3 *SD*s; see Fig. 1c) and were analyzed with a 2 × 2 repeated-measures ANOVA. Main effects were significant—agent: *F*(1, 29) = 126.8, *p* < .001, η_p_^2^ = .81; condition: *F*(1, 29) = 89.80, *p* < .001, η_p_^2^ = .76. The interaction was not significant, *F*(1, 29) = .28, *p* = .60, η_p_^2^ = .01. Participants responded more slowly in the human condition and in the experimental condition. Overall, participants made more errors in the experimental trials with the robot director, consistent with the humanization hypothesis.

### Interim summary of Experiment 1

To summarize, using the director task, we found that participants’ error rate was lower, but their RT was longer when interacting with a human agent in the experimental condition, where a competitor object could interfere participants’ choices. This result suggested that people might prefer to spend more time in taking a human agent’s perspective so as to achieve lower error rate, thus it supported our humanness account for VPT in the comparison between human and robot. In Experiment 2, we would like to use the same task to test the humanness hypothesis in the intergroup context.

## Experiment 2

Our second study uses a more subtle manipulation of humanization, building on evidence that people tend to dehumanize out-group members (Haslam, 2006; Leyens, Demoulin, Vaes, Gaunt, & Paladino, 2007; Vaes et al., 2004). Our method closely matched Experiment 1, but we used two agents with human appearance and added a minimal group manipulation to make participants feel as though they were in the same group as one and in a different group from the other.

### Method

#### Participants and materials

**Fig. 2:**
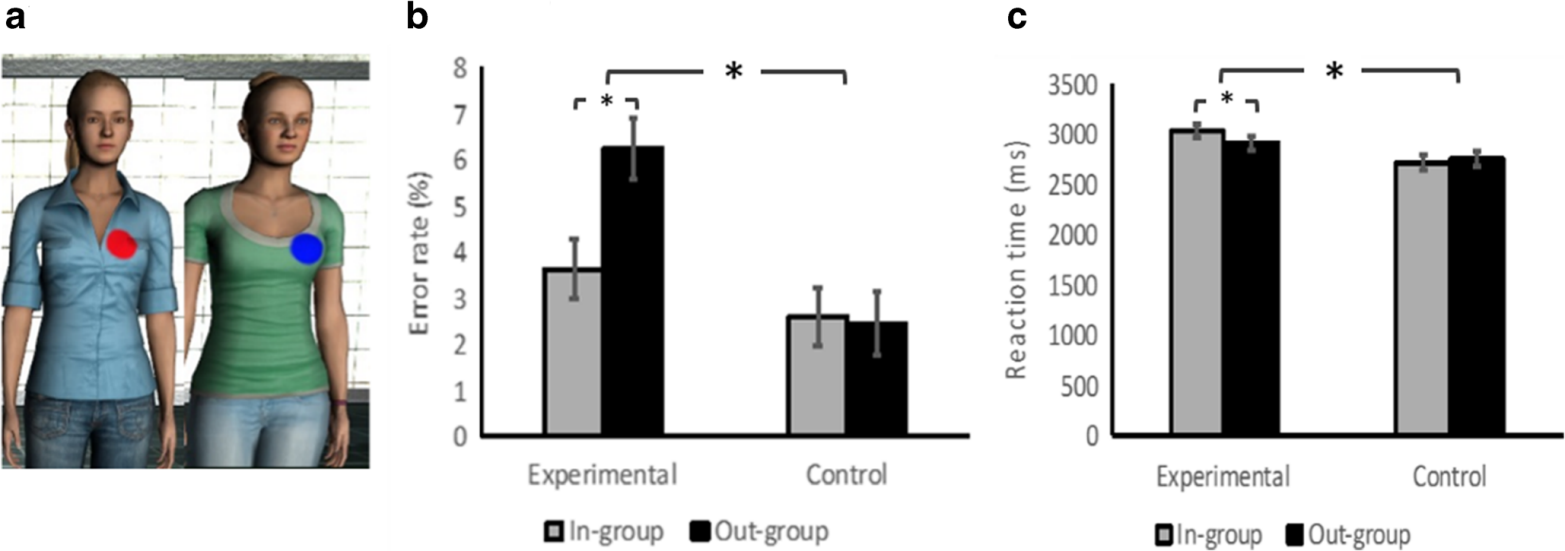
Experiment 2. **a** In-group and out-group figures. **b** Error rates for Experiment 2. **c** Reaction times for Experiment 2. Error bars indicate the standard errors. * *p* < .05, ** *p* < .01

Female participants (mean age = 23.0 years, *SD* = 2.88 years) were recruited and gave written informed consent for the study, which was approved by the ICN ethics committee (ICN-AH-PWB-3-3-14a). The sample size (*n* = 32) was predefined based on a power analysis showing this provides at least 80% power to detect a medium effect size (Cohen’s *f* = 0.25). We used the same software and tasks as previously. The only change was that we used two distinct virtual agents and voices recorded from two different native British speakers for the director task instructions (see Fig. 2a).

#### Procedure

Participants came to the lab individually and gave informed consent. Their first task was a dot-counting task, used to set up the in-group/out-group manipulation (Howard & Rothbart, 1980). This was implemented in Cogent/MATLAB and presented on a laptop. On each trial, participants saw a black screen with 50–250 white dots for 2 seconds and were asked to judge the number of dots. After 10 trials, all the participants labelled as “underestimators” and were given a blue sticker to place on their clothes as an indication of their group membership. They then saw pictures of the two virtual agents (Kate and Jess) and learnt that Kate was an underestimator whereas Jess was an overestimator (counterbalanced across participants). This was reinforced by red and blue stickers on Kate and Jess’s clothes (see Fig. 2a).

Participants then put on the HMD and completed the familiarization and the director task in just the same way as in Experiment 1; the only change was the appearance and voice of the virtual agents. After the trials were completed, all participants completed the same questionnaires as in Experiment 1, with an additional four questions to check the effectiveness of the group manipulation (see Supplementary Info. S2).

### Results

Error rates were analyzed using a 2 × 2 repeated-measures ANOVA, with group (in-group, out-group) and condition (experimental, control) as within-subjects factors (see Fig. 2b). Holm corrections were used for post hoc multiple comparisons. There was a main effect of condition, *F*(1, 31) = 10.85, *p* < .001, η_p_^2^ = .26, with more errors in the experimental condition but no main effect of group membership, *F*(1, 31) = 3.90, *p* = .06, η_p_^2^ = .11. Critically, there was a reliable interaction between condition and group, *F*(1, 31) = 4.49, *p* = .04, η_p_^2^ = .13. In the post hoc analysis, in the experimental trials, participants made more errors in the out-group condition than in the in-group condition, *t*(31) = 2.93, *p* = .01, whereas there was no difference between conditions in the control trials, *t*(31) = .15, *p* = .88. Again, a bootstrap analysis confirmed these results can also be seen without assuming normal distributions.

The mean reaction times were calculated from correct responses for each participant after excluding extreme values (±3 *SD*s; see Fig. 2c) and analyzed with a repeated-measures ANOVA as before. There was a significant main effect of condition, *F*(1, 31) = 20.75, *p* < .001, η_p_^2^ = .40, with longer reaction times for the experimental condition, but no effect of main effect of group, *F*(1, 31) = 2.96, *p* = .10, η_p_^2^ = .09. There was an interaction between condition and group, *F*(1, 31) = 5.37, *p* = .03, η_p_^2^ = .15. According to the post hoc analysis, in the experimental trials, participants responded more slowly to the in-group than the out-group, *t*(31) = 3.87, *p* < .001, whereas there was no difference between conditions in the control trials, *t*(31) = 1.44, *p* = .16.

## Experiment 3

**Fig 3:**
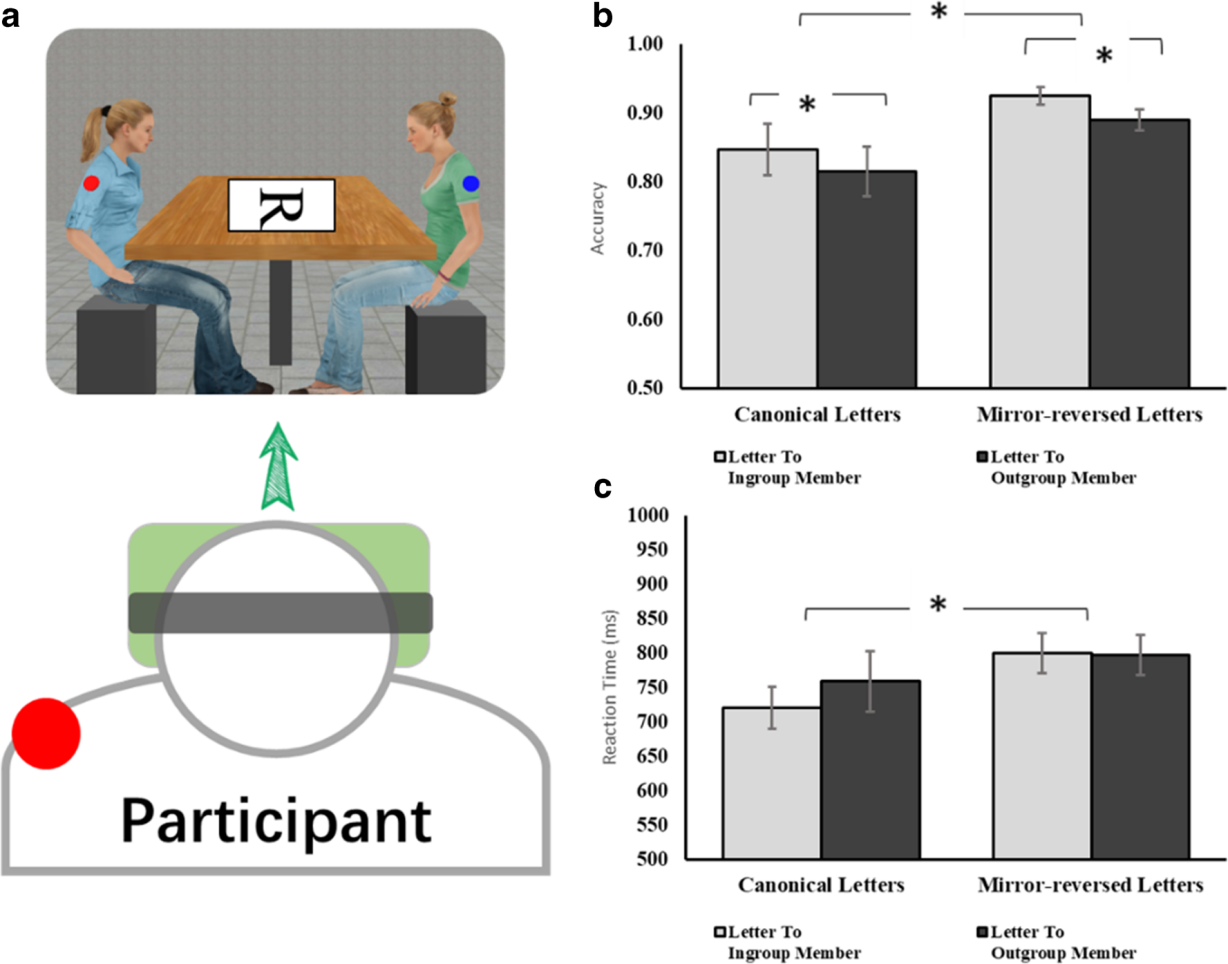
Participants wearing a VR headset see two agents sitting at a table **a** with the letter stimuli appearing in the centre of the table (the white rectangle and its frame here are to highlight the letter. In the experiment, letters were directly presented on the table without any background). The agents have red/blue stickers to indicate if they are over or under estimators, and the participant also has a sticker for group membership. Results are shown in terms of accuracy **b** and reaction time **c.** Error bars indicate the standard errors. * *p* < .05, ** *p* < .01

**Fig. 4:**
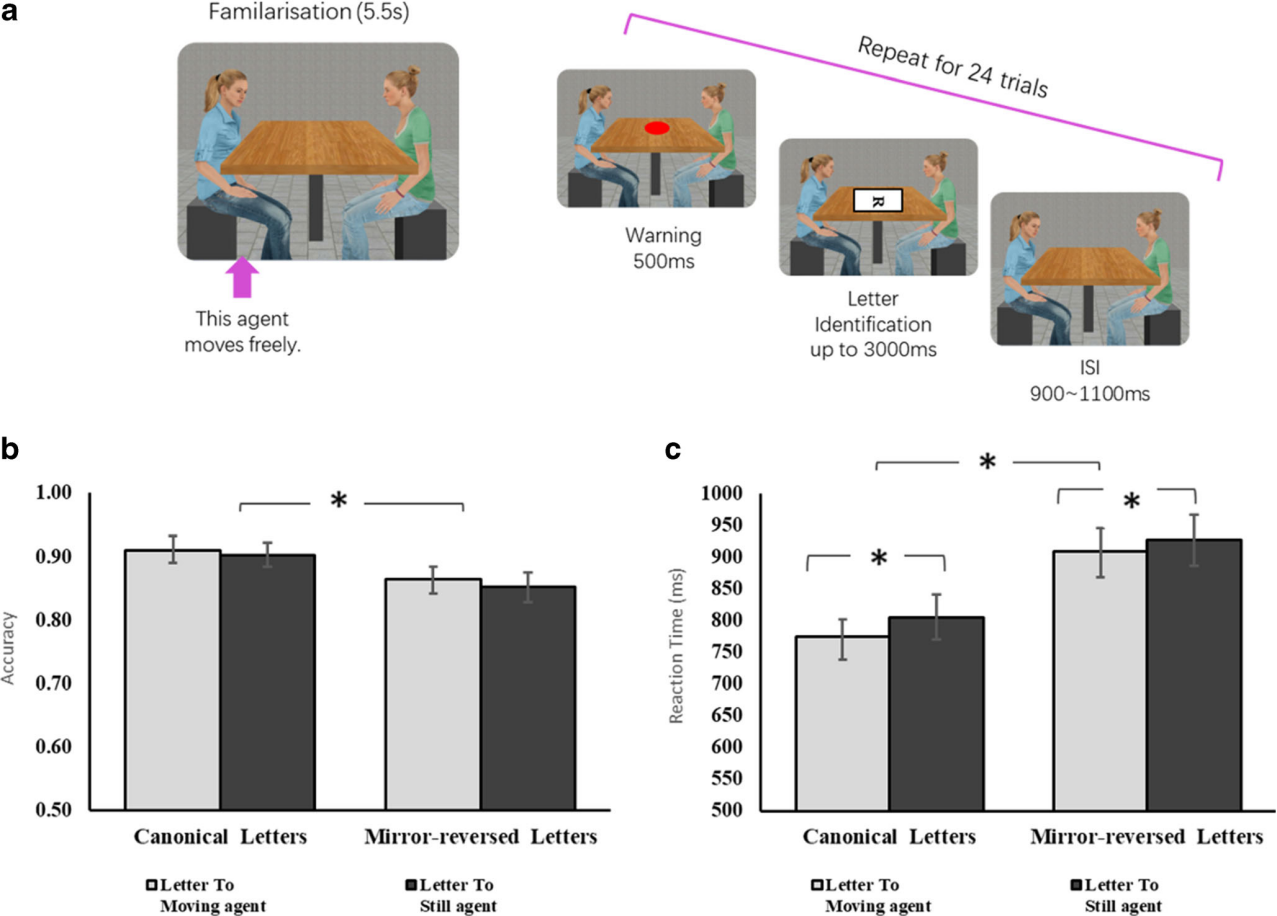
In the VR, participants saw the agents moving freely for a 5.5-seconds familiarization period and then completed 24 trials **a**. Here, the agent in blue moves naturally while the agent in green is a statue. The white rectangle and its frame here are to highlight the letter. In the experiment, letters were directly presented on the table without any background. Results are shown in terms of accuracy **b** and reaction time **c**

Experiments 1 and 2 support our hypotheses that typical adults show less spontaneous VPT when encountering a non-human agent or a dehumanized out-group member. However, in Experiment 3, participants still encountered only one agent at a time. If the effect of humanization is strong, it should also be seen in contexts where two agents are present, and it should generalize to other tasks. Ward et al. (2019) introduced a new social mental rotation task, in which participants must judge whether a rotated letter on a table is a normal or mirror-reversed *R*. Critically, if another person was present in the scene, participants were faster to perform the task when the letter was oriented towards the other person. Thus, Ward et al. claim that participants spontaneously take other’s perspectives. Here, we developed a similar task using two agents who have different perspectives (see Figs. 3a and 4a). For Experiment 3, we used the minimal group manipulation to establish in-group and out-group agents, and then tested whether participants prefer to take the perspective of the in-group member when the two perspectives are in conflict.

### Method

#### Participants

We calculated the sample size using G*Power 3.0 based on the results of our pilot study (see Supplementary Info. S3). To achieve an effect size of 0.25 (Cohen’s *d*) and at least 80% power on a .05 significant level, 36 right-handed participants were recruited in this experiment (23 females, mean age = 25.4 years, *SD* = 4.90 years). Participants were required to have the Latin alphabet as the basis of their first language and normal or normal-to-corrected vision. Payment and recruitment details are the same as in the previous two experiments.

#### Materials and VR setup

VR setting was created by in Vizard 5.0 (Worldviz, USA). Participants wore the Oculus Rift DK2 and saw a virtual room where a wooden table was placed with two female agents sitting on the left or right (see Fig. 3a). Both agents had a European appearance and moved according to the Vizard ‘quiet sitting’ animation with breathing and small movements; the agent’s head was oriented so that they were looking towards the centre of the table where the stimuli appeared. We used four asymmetric letters (*F, R, P* and *G*) in Times New Roman font as our stimuli.

In the beginning of each block, the two agents sat naturally in the virtual room by the table for 5.5 seconds to let participants adapt to the scene and become familiar with the positions of the in-group and out-group agents. A red dot then appeared at the centre of the table for 500 ms, indicating the upcoming letters. On each trial, a letter appeared in the centre of the table, orienting towards one of the agents or towards the participant. Participants were instructed to press key ‘J’ if the letter was canonical and ‘F’ if it was mirror-reversed. After the key hit, the letter disappeared and there was an interstimulus interval of 900–1,100 ms before the next trial began. The room and agents all remained visible during the interstimulus interval, to maintain the feeling of being in a real location with real people.

Each participant completed two blocks with 48 trials in each of them and a short break between. In each block, there were 16 trials the letters facing towards the in-group agent, 16 trials towards the out-group agent and another 16 toward the participant. By using this manipulation, we endow equal importance to both the agents’ and the participant’s egocentric perspectives. Letter-type (canonical or mirror-reversed) was balanced within each direction. Trials were presented in random order.

#### Procedure

Participants gave written informed consent to take part. We used the same dot-counting task as in Experiment 2 to set up the minimal group manipulation. After the dot-counting task, participants were given a coloured sticker with which to mark themselves as an overestimator/underestimator and were introduced to the two agents (named Lucy and Ellie). Participants were told the agents were real participants who completed the same task on a previous day, and one of them was an overestimator and the other an underestimator. Then they were instructed to put on the HMD and get familiar with the room. During the experiment, the two agents wore coloured stickers indicating their group. Both the participant’s and the agents’ label as an underestimator/overestimator were balanced across participants. Agents’ sitting positions were balanced across blocks. Participants completed the 96 trials of the social mental rotation task, and then filled in a questionnaire about their strategies and their attitude towards the two agents (see Supplementary Info. S4). The whole study took about 20 minutes to finish.

### Results

A 2 × 2 repeated-measures ANOVA was used to analyze accuracy for all trials and reaction time for correct trials after excluding extreme values (±3 SDs), with letter-type (canonical; mirrored-reversed) and agent-group (in-group, out-group) as within-subjects factors. For accuracy, congruent to our hypothesis, a significant main effect was found for agent (*F* (1, 35) = 8.24, *p* = .007, η_p_^2^ = .19). Participants performed better when the letters were facing towards the in-group agent. There was also a significant main effect for letter-type (*F* (1, 35) = 4.23, *p* = .047, η_p_^2^ = .11), with mirror-reversed letters processed better than canonical letters (Fig. 3b). For RT, a significant main effect was observed for letter-type (*F* (1, 35) = 5.48, *p* = .025, η_p_^2^ = .14), with canonical letters processed more quickly than mirror-reversed versions (Fig. 3c). No other main effect or interaction reached significant level.

## Experiment 4

Study 3 showed that, when participants encounter two people with conflicting perspectives, they prefer to take the perspective of the in-group member and their task performance improves for items oriented towards the in-group member. This is the first study to examine perspective selection in multi-agent perspective-taking and demonstrates a humanization effect, paralleling the results from the Director task in Expt. 2. For our final study, we wanted to explore the effects of humanization with a more subtle manipulation. Thus, we test if participants prefer to take the perspective of an agent who moves like a human, compared to one who is rigid like a statue.

### Method

#### Participants

36 right-handed participants were recruited from two UCL-associated psychology databases (25 females, Mean age = 21.9, *SD* = 3.61). Our requirements and payments for the participants remained the same as in Expt. 3.

#### VR stimuli

The VR setup here was closely modelled on Expt. 3, with the following changes. We did not use the minimal group manipulation, but instead contrast a moving virtual agent who performs natural human actions with one who is rigid like a statute. To avoid having agents switch between ‘moving’ and ‘non-moving’ roles, we used two female agents in blocks 1 and 2, and then two male agents in blocks 3 and 4. The position of the moving agent (left or right) was balanced across the two halves and which agents moved were balanced across participants. The moving agent performed natural seated actions, such as moving the head and torso and looking around, while the still agent was like a statue with no motion. Both agents had a neutral facial expression.

Each block of the task began with a 5.5 seconds familiarization period where participants could look around the virtual space. During this time, the moving agent showed some large movements (turning her head, shifting posture) while the static agent remained rigid. When the trials were about to begin, the moving agent oriented his/her head and body towards the table and showed only the small ‘quiet sitting’ movements, as used in Expt. 3. Again, the static agent remained rigid.

#### Procedure

Participants gave written consent and had time to get familiar with the VR before the task began. They completed four blocks of trials with 24 trials per block. As before, each block began with a 5.5s familiarization period where one agent moved and the other was static, followed by the trials (Fig. 4a). In each trial, one letter appeared in the centre of the table and participants had to judge if it was canonical or mirror-reversed. In each block, there were eight trials letters are facing towards the participant, eight towards left and eight towards the right. Letter type was all balanced within each direction and all trials were presented in random order. After the computer-based task, participants were asked to fill out two short questionnaires in which we checked their preference for the two agents and their personalities (see Supplementary Info. S5).

### Results

A 2 × 2 repeated-measures ANOVA was applied to analyze accuracy for all trials and reaction time for correct trials after excluding extreme values (±3 *SD*s), with letter-type (canonical; mirror-reversed) and agent (moving, still) as within-subjects factors.

For accuracy, there is a significant main effect for letter-type, *F*(1, 35) = 4.32, *p* = .045, η_p_^2^ = .11, with participants performed better on canonical letters than on mirror-reversed versions (see Fig. 4b). No main effect or interaction was found related to agent.

For RT, we found a significant main effect for letter-type, *F*(1, 35) = 45.37, *p* < .001, η_p_^2^ = .57, and normal letters were processed more quickly than its mirrored versions. Importantly, we observed a significant main effect for agent, *F*(1, 35) = 4.23, *p* = .047, η_p_^2^ = .11: Participants responded quicker when letters were presented to the moving agent. No other main effect or interaction effect reached significant (see Fig. 4c).

## General discussion

The aim of this study was to determine if humanization of an agent modulates performance on a visual perspective-taking task in contexts with a single agent or with multiple agents. Using virtual reality, we were able to present our moving stimuli in a 3D format with a context that remains constant over all the trials, giving greater ecological validity than typical lab studies. We used two different VPT tasks—the director task and the social mental rotation task, and two different manipulations of humanization—an in-group/out-group manipulation and a human/robot-statue manipulation. In all cases, results showed stronger VPT effects for the in-group and human agents, compared with the out-group, robot, or statue agents. These results indicate that our initial perception of another agent as human or not plays a critical role in determining our propensity to take the perspective of the other agent.

These results add to our understanding of how perspective-taking processes relate to other aspects of cognition. Previous cognitive studies of VPT have emphasized the rapid, even automatic nature of this process (Furlanetto, Becchio, Samson, & Apperly, 2016; Samson et al., 2010; Surtees & Apperly, 2012; Michael, et al., 2018), or have tried to show modulation of VPT by adding dual tasks or changing motivation (Bukowski & Samson, 2016; Cane, Ferguson, & Apperly, 2017; Todd, Simpson, & Cameron, 2019). Such studies suggest that VPT is relatively impervious to these manipulations. In contrast, our data show that changes in the perception of the agent can change the propensity to engage in VPT. That is, we believe that our data can be understood in terms of two consecutive cognitive processes. First, participants must humanize one agent in the scene, based on a range of criteria. When a human agent is detected, participants can then engage in the process of taking that person’s perspective. This can involve inhibiting information about what the agent cannot see (director task) or enhancing perspectives on what the agent can see (social mental rotation task). Overall, we suggest that the humanization process can act as a gateway to perspective-taking, which could then proceed rapidly and spontaneously.

Here, we used several different manipulations of the humanness of our computer-generated agents. In Experiments 1 and 4, we controlled perceptual information about the agent in terms of its appearance as a robot or its movement patterns (natural v. statue). In Experiments 2 and 3, we controlled top-down information about the agent with a well-established minimal group manipulation. All of these changes had the same impact: Decreasing an agent’s humanness reduced the propensity of participants to take the agent’s perspective. Thus, we interpret all four studies under the general framework of humanization and dehumanization, whereby people tend to categorize others into in-group/out-group and affords out-group individuals less positive human essence compared with in-group individuals (Boccato, Cortes, Demoulin, & Leyens, 2007; Loughnan & Haslam, 2007). It seems that robots and statues are treated similarly to dehumanized individuals in our studies, and our results show how processes of humanization are critical to even basic aspects of social cognition like VPT.

Our results revealed several different findings compared with previous studies. First, although both our study and Savitsky et al. (2011) study used the director task to test whether varying the identity of the director could impact VPT, the result patterns seem to be opposite. In Savitsky et al.’s (2011) study, participants made more errors when the director was a friend rather than a stranger, but here the performance for a more humanized agent was generally better. We consider this discrepancy may be because participants overinterpret the instruction of a friend compared with that of a generic in-group agent (i.e., as a friend is closer than an in-group stranger, participants might expect they were aware of the presence of a competitor object and were referring to it when giving the instruction). Similarly, although we found that the in-group relationship can boost VPT in the social mental rotation task, Todd and colleagues (2011) reported that it can impair the task performance when participants needed to use perspective-taking to walk of out a maze. Such difference in results may be due to the mutual perspective-taking process in Todd’s task (i.e. when the guider of the pair trying to take the walker’s perspective to give the correct instruction, the walker was also trying to use perspective-taking to overcome the ambiguity of the instruction). Such a mutual perspective-taking process may result a cost in the total time for completing the task.

Our current study manipulated the humanness levels of different virtual agents; however, such virtual agents might not be fully humanized. We believe future studies on perspective selection might be able to use real people (Freundlieb et al., 2016, 2018). Another point worth noting is that humanization has an impact on different indices in Experiments 3 and 4. Participants responded more accurately in the intergroup context, but responded faster when exposed to moving and statue agents. We consider that this discrepancy may due to a speed–accuracy trade-off, as results from our pilot study of the intergroup setting did reveal faster responses when canonical letters are oriented towards the in-group agent.

This study focused on an ecological question by asking how people select perspective in real life, considering we often encounter multiple perspectives at the same time. Results emphasize the modulation role of humanization and suggest our social cognitive capacities might be target specific. With the digitization of modern society, we believe such results are valuable for investigating how people interact with various virtual agents, robots, or avatars of our close others in the future. Industrial designers may also consider the way to humanize such agents in order to promote social interaction in the digital world. Future studies may consider testing such findings in real the world, and measuring how such propensity to engage in perspective-taking varies in clinical populations.

## Supplementary Information

ESM 1 (DOCX 1045 kb)
