# Supplementary material for: Taking the perspectives of many people: Humanization matters
Source: Psychon Bull Rev. 2020 Dec 14;28(3):888–97. doi: 10.3758/s13423-020-01850-4 (PMC8219553; doi:10.3758/s13423-020-01850-4)
Supplement: Supplementary file 1 — (DOCX 1045 kb) [file 13423_2020_1850_MOESM1_ESM.docx]

**Supplementary Information**

**S1. Task layout for the director task in Expt 1 and Expt 2.**

Participants completed the director task in a virtual factory (shown here with two human agents) with one human or robot agent. In the factory, a conveyor belt (green line at the bottom) carried stimuli from one machine (on the left) to another (on the right). The stimuli were a 3x3 set of shelves with 3 shelves occluded from the Director’s view. On each trial, the shelves first emerge on the conveyor, then the director walks into the room and looks at the shelves. The director instructs the participant to take an object (e.g. *Take the small dice*) and the participant must move a hand icon using the mouse to click on the correct object. On experimental trials, the participant must work out that the director cannot see the smallest (black) dice thus she is referring to the red dice. On control trials, there is no ambiguity. After the participant selects an object, that item disappears from the shelves and the shelves move away on the conveyor belt. The director also walks out so that the scene is ready to start the next trial.


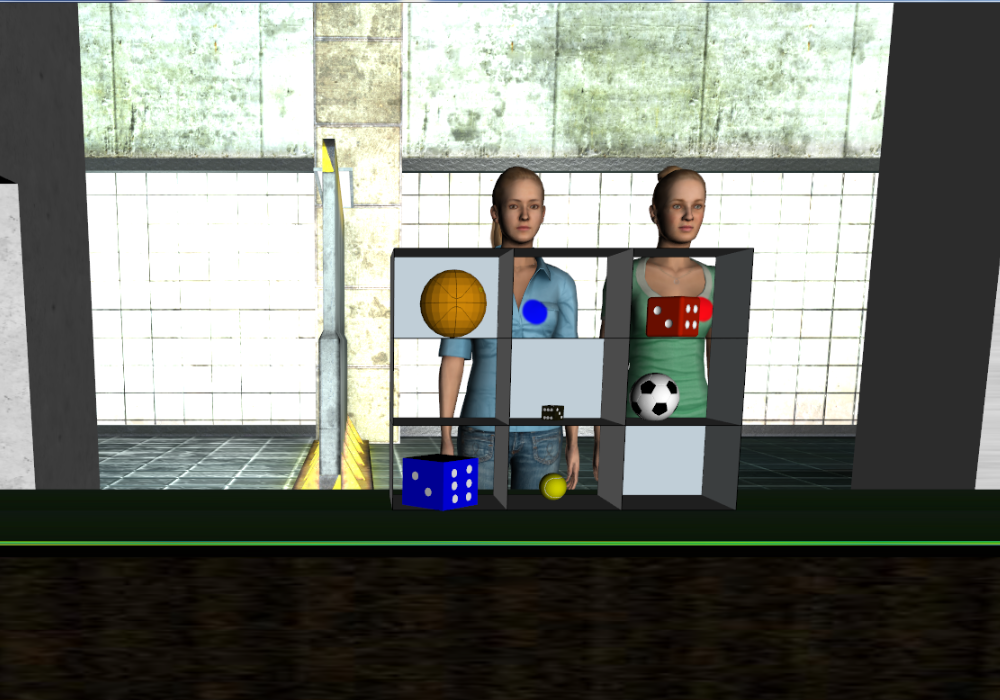


**S2. Questionnaire results on Experiment 1 & 2**

Participants in Expt. 1 and 2 completed a questionnaire after the task to assess their reactions to the agents in VR. The questions and mean responses to each agent are given below. In Expt. 1, results show that participants reported the human was more real and likeable than the robot and that they were more motivated to respond to the human. In Expt. 2, results revealed that the ingroup agent was more likeable; participants felt closer to her and reported being more motivated to respond to her. These results confirm that the manipulations of humanness/group identification were successful in both studies.

**Table 1.** Questions asked in the post-study questionnaire and the responses given (on a 7 point Likert scale anchored with strongly disagree / strongly agree).

| **Experiment 1: Question** | **Human** | **Robot** | **Difference** |
| --- | --- | --- | --- |
| The lady/robot seemed very real to me | Mean 4.73  (SD 1.34) | Mean 3.13  (SD 1.59) | t = 6.87,  p < .001 |
| I responded to her/it as if she/it were real | Mean 5.43  (SD 1.61) | Mean 4.90  (SD 1.90) | t = 2.64,  *p =* .01 |
| My thoughts in relation to her/it were as if she/it were real | Mean 4.77  (SD 1.76) | Mean 4.72  (SD 1.89) | t = 1.81,  *p =* .08 |
| I like her/it very much | Mean 4.50  (SD 1.26) | Mean 3.57  (SD 1.48) | t = 3.45,  *p =* .002 |
| In spite of my knowledge that she/it wasn’t real, I found myself behaving as if she/it were real | Mean 5.13  (SD 1.52) | Mean 4.73  (SD 1.67) | t = 3.03,  *p =* .01 |
| I had motivation to understand what she/it said | Mean 6.03  (SD 0.75) | Mean 5.60  (SD 1.17) | t = 3.07,  *p =* .01 |
| The lady/robot was very different from me | Mean 4.83  (SD 1.46) | Mean 6.67  (SD 0.60) | t = 6.86,  *p* < .001 |
|  |  |  |  |
| **Experiment 2: Question** | **Ingroup** | **Outgroup** | **Difference** |
| The lady seemed very real to me | Mean 4.22 (SD 1.74) | Mean 3.94 (SD 1.61) | t = 1.61,  *p =* .12 |
| I responded to her as if she/is were real | Mean 5.41 (SD 1.52) | Mean 5.09 (SD 1.86) | t = 1.58,  *p =* .13 |
| My thoughts in relation to her were as if she/it were real | Mean 4.56 (SD 1.85) | Mean 4.03 (SD 1.15) | t = 1.64,  *p =* .11 |
| I like her very much | Mean 4.53 (SD 1.24) | Mean 4.03 (SD 1.15) | t = 2.23,  *p =* .03 |
| In spite of my knowledge that she wasn’t real, I found myself behaving as if she were real | Mean 5.19 (SD 1.73) | Mean 4.91 (SD 1.86) | t = 1.79,  *p =* .08 |
| I had motivation to understand what she said | Mean 6.16 (SD 1.08) | Mean 5.91 (SD 1.25) | t = 2.49,  *p =* .02 |
| The lady was very different from me | Mean 4.66 (SD 1.49) | Mean 5.00 (SD 1.57) | t = 1.73,  *p =* .09 |
| I feel close to underestimators / overestimators | Mean 4.19  (SD 1.47 ) | Mean 3.38  (SD 1.40) | t = 2.60  *p =* .01 |
| Kate / Jess is an underestimator / overestimator | 100% correct | 100% correct | ns |

**S3. Pilot Study for Experiment 3**

To obtain the appropriate sample size and experimental stimuli for the 2-person social mental rotation task, we first conducted a pilot study. We report the results of this pilot in full here for completeness.

*Participants*

29 (18 females, aged 23.5±3.2) right-handed participants took part in this experiment; however, five participants were excluded from further analysis because the post-experiment survey showed that they detected the purpose of our study. This resulted in a final sample size of 24 participants. Participants were recruited from two UCL-associated psychology databases and were required to have the Latin alphabet as the basis of their first language and normal or normal-to-corrected vision. Participants were paid based on a rate of £7.5 per hour.

*Materials & VR setup*

VR setting was created by in Vizard 5.0 (Worldviz, USA). Participants wore the Oculus Rift DK2 and saw a virtual room where a wooden table was placed with two female agents sitting on the left or right (Figure *2*). Both agents had a European appearance and moved according to the Vizard ‘quiet sitting’ animation with breathing and small movements but no head turns. We added a small rotation to agents’ heads and neck so both agents gazed towards the table as if they were looking at the stimuli. On each trial, one letter was presented in the middle of the table in Arial font from the set F, R, P, G, and Q. After the study was complete, we realised that the letter Q was an ambiguous stimulus without a definite orientation. For example, a normal Q viewed from the participant’s location appeared very similar to a reversed Q viewed by a person sitting to the right of the participant. This lead to high error rates for Q and so we decided to remove all the Q trials and present methods and results here only with the rest four letters (R, F, P, G).

Each block of trials began with the two agents sitting naturally in the virtual room to let participants explore the scene for 5.5 seconds and see who was located where. Then a red dot appeared on the table for 500 ms to draw attention from the participants and the trials then began. In each trial, a letter appeared in the centre of the table, oriented towards one or the other agent or to toward/away from the participant. Participants were instructed to press key ‘J’ if the letter was normal and ‘F’ if it was a mirrored as fast as possible. After the keyhit, the letter disappeared and there was an ISI of 900-1100ms before the next trial began. Note that the room and agents all remained visible during the ISI, to maintain the feeling of being in a real location with real people.

For letters F, R, P, and G, trials are organized in two blocks with a total of 72 trials. The two agent’s sitting positions were balanced between blocks. In each block, there were 32 trials where letters would face towards one of the agents, besides, there were two trials where letters would face to the participants and another two face away from the participants. We added these four distracting trials make it harder for participants to detect the purpose of the study. Between the two blocks, there was a break of at least 1.5s then participants could press the ‘space’ when they were ready to resume.

*Procedure*

Participants gave written consent to take part. We used the same dot-counting task in Experiment 2 as the minimal group manipulation. After the dot-counting task, they were given a coloured sticker to mark them as an over-estimator/underestimator and were introduced to the two agents (named Lucy and Ellie). Participants were told the agents were real participants who completed the same task on a previous day, and one of them was an overestimator and the other an underestimator. Then they were instructed to put on the HMD and get familiar with the room. During the experiment, the two agents would wear coloured stickers according to their roles, and their roles were balanced across participants. Their sitting positions were balanced across blocks. The whole study took about 20 minutes to finish.

**Results**

A 2×2×2 repeated-measurement ANOVA was applied to analyze accuracy for all trials and reaction time (RT) for correct trials after excluding extreme values (± 3 SDs), with letter-direction (left; right), letter-type (canonical; mirror-inverted) and agent (ingroup, outgroup) as within-subjects factors. For accuracy, no significant main effect or interactions were found related to the agent factor. There was a significant main effect for letter-direction, with more accurate responses for letters towards the right (*F* (1,23) = 4.81, *p* = .039, η_p_^2^ = 0.17). As all our participants were right-handed, this result may reflect an effect of handedness.

For RT, a significant main effect was observed for letter-type (*F* (1, 23) = 26.52, *p* < .001, η_p_^2^ = 0.57), with canonical letters being processed more quickly than its mirrored versions. A significant interaction was found for letter-type×agent (*F* (1, 23) = 7.36, *p* = .012, η_p_^2^ = 0.24), when canonical letters are towards an ingroup member, participants made faster responses (*t* = -2.62, *p* = .015), but this was not the case for mirror-inverted letters (*t* = 0.86, *p* = .400).


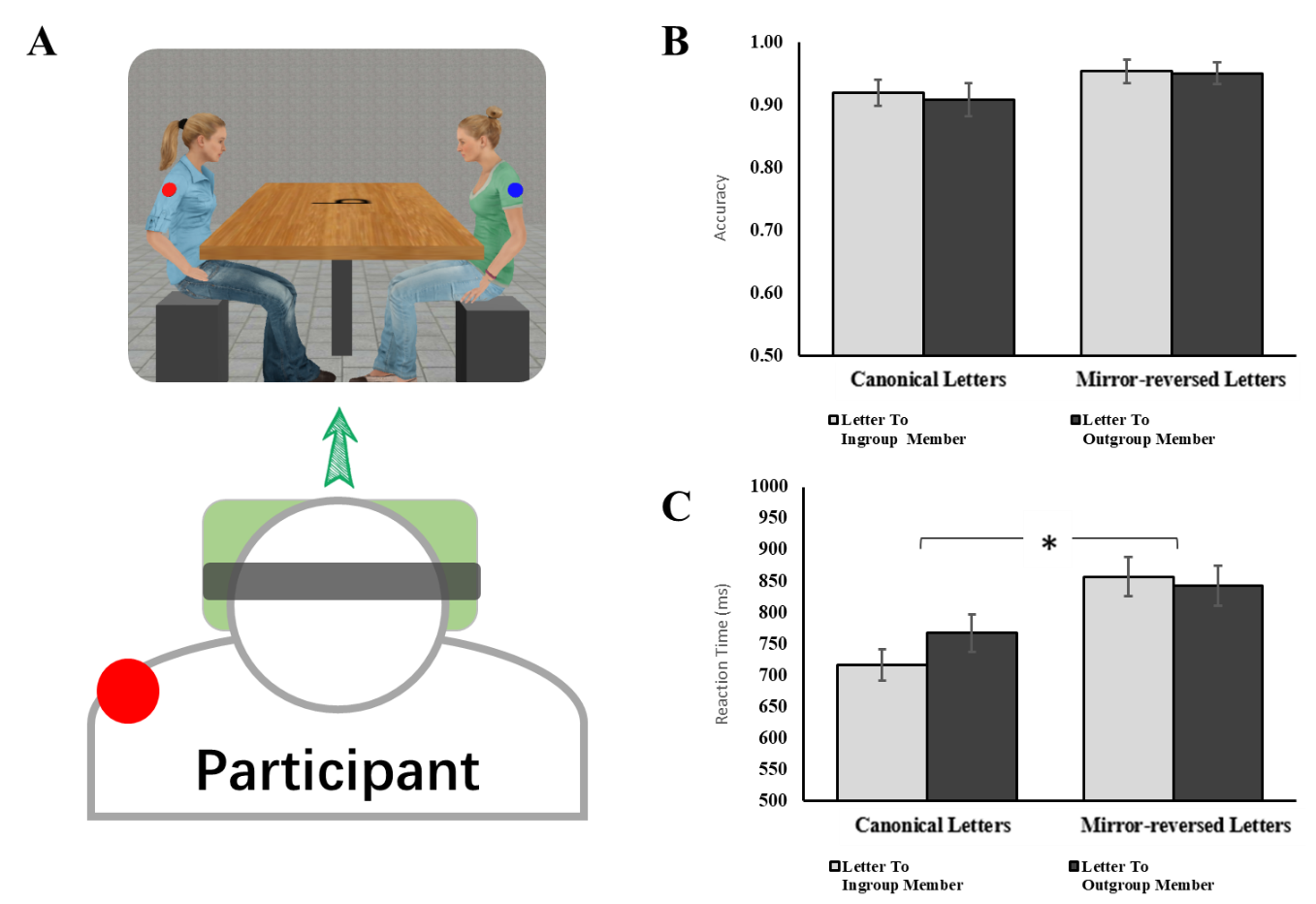


Figure 2. An illustration of the VR setting in the Pilot study (A); Results on accuracy of recognising letters F, R, P and G (B); Results on reaction time (ms) of recognising letters F, R, P and G (C).

**Discussion**

Results from our pilot study support that the current paradigm is effective to investigate perspective selection in multi-perspective scenarios. Being exposed simultaneously to perspectives both from an ingroup and an outgroup member, participants were able to recognise normal letters faster when the letter oriented towards an ingroup member, indicating they have a stronger propensity to take the inroup member’s perspective. These results also showed that our two-person mental rotation task is effective to test perspective selection. We then used the results from the pilot study to calculate the proper sample size for our formal study in G*power 3.1. To achieve an effect size of 0.8 on a .05 significant level, G*power suggested a sample size of 35. In the end, we decided to test 36 participants for Experiment 3 & 4.

**S4. Questionnaire Results from Experiment 3**

**Method**

To test if participants explicitly identified more with the ingroup agent, we also include questionnaire methods to measure participants’ expressed identification and interactive motivation with the two different agents.

Two questionnaires were used here. They were the relatedness subset from the Intrinsic Motivation Inventory (IMI) (McAuley, Duncan, & Tammen, 1989), and the Group Identification Scale used in many group identification studies (Doosje & Spears, 1995; Hertel & Kerr, 2001). The latter includes four different statements covering the general, cognitive, evaluative and affective aspects of group identification. Where necessary, we changed the expression a bit so it sounds more natural to describe the agents in the current task. Participants were asked to rate on a 1 (not at all) to 7 (very true) points scale about how they feel about the ingroup and the outgroup agent in the task.

The questionnaire items are:

*IMI relatedness subscale:*

- I felt really distant to this lady. (R^[[1]](#footnote-1)^)
- I really doubt that this lady and I would ever be friends. (R)
- I felt like I could really trust this lady.
- I’d like a chance to interact with this lady more often.
- I’d really prefer not to interact with this lady in the future. (R)
- I don’t feel like I could really trust this lady. (R)
- It is likely that this lady and I could become friends if we interacted a lot.
- I feel close to this lady.

*Group Identification Scale:*

- I identify with the group which this lady belongs.
- I see myself as member in the group which this lady belongs.
- I feel stronger link to this lady.
- I’m glad to be a member in the group which this lady belongs.

Apart from these two questionnaires targeting on group identification, we also asked participants to fill up questions used in Experiment 1 & 2, to check participants’ impression on the two agents.

**Analysis & Results**

For each participant, we calculated the total score from all eight items from the IMI relatedness subscale and that for all four items of the Group Identification Scale for further exploratory analysis. We also compared participants’ scores for the ingroup/outgroup agents on each question from the survey we used in Expt. 1&2.

Paired-sample t-tests failed to show any difference in each of the measurements we included. We summarized all results in Table. 2.

**Table 2.** Questions asked in the post-study questionnaire in Expt. 3 and the responses given (on a 7 point Likert scale anchored with strongly disagree / strongly agree).

| **Experiment 3: Measurements** | **Ingroup** | **Outgroup** | **Difference** |
| --- | --- | --- | --- |
| Intrinsic Motivation Inventory (IMI) | Mean 28.19  (SD 8.60) | Mean 26.88  (SD 7.81) | t = 0.65,  *p =* .518 |
| Group Identification Scale | Mean 12.25  (SD 5.56) | Mean 11.36  (SD 5.90) | t = 0.85,  *p =* .402 |
| The lady seemed very real to me | Mean 2.75  (SD 1.92) | Mean 2.39  (SD 1.66) | t = 1.33,  *p =* .191 |
| I responded to her as if she/is were real | Mean 2.50  (SD 1.86) | Mean 2.50  (SD 1.71) | t < 0.01,  *p* > .999 |
| My thoughts in relation to her were as if she/it were real | Mean 2.56  (SD 1.87) | Mean 2.53  (SD 1.93) | t = 0.135,  *p =* .893 |
| I like her very much | Mean 3.42  (SD 1.44) | Mean 3.31  (SD 1.62) | t = 0.412,  *p =* .683 |
| In spite of my knowledge that she wasn’t real, I found myself behaving as if she were real | Mean 2.75  (SD 1.93) | Mean 2.50  (SD 1.87) | t = 1.103,  *p =* .278 |
| The lady was very different from me | Mean 4.33  (SD 1.37) | Mean 4.25  (SD 1.50) | t = 0.255,  *p =* .800 |

**Discussion**

Our questionnaire results showed that participants did not explicitly identify more with the ingroup agent, and also showed no stronger extrinsic motivation to engage in future interaction with the ingroup agent. These findings differed from previous results from Experiment 2, where participants were more affliated with and preferred the ingroup member. Such discrepancy might due to distinct features of the two tasks, that in the Director Task, participants needed to process the agent’s verbal instructions and act upon that, however the social mental rotation task is non-verbal and agents were mere observers, a lack of direct social contact may hinder participants from forming explicit impressions onto the agents.

**S5. Questionnaire Results from Experiment 4**

**Method**

Participants were given two questionnaires to fill out after completing the social mental rotation task. The first was composed of a series of questions targeting participants’ attention to the task and their preference towards the agents. They were instructed to rate how much they liked each agent (on a 1-6 Likert scale), to recall which two agents were moving, to guess the purpose of the task and to report the strategy they used in the experiment. The second was the Ten Item Personality Inventory (TIPI) (Gosling et al., 2003), and was included to test if participants’ propensity to take the moving agent’s perspective is correlated with their personality. The TIPI contains two items for each of the five dimensions in the Five Factor model, and they are extraversion, agreeableness, conscientiousness, emotional stability, and openness to experience (e.g. ‘I see myself as reserved and quiet’). Participants were required to indicate the extent to which they agree to each statement on a seven-point Likert scale distributing from ‘strongly disagree’ to ‘strongly agree’. We then calculated the differential scores for accuracy and reaction time between the moving and static agent’s conditions, and tested their potential correlations with these psychological traits. Meanwhile, we calculated the differential likability score between the two agents by subtracting participants’ likeability scores towards the static agents from that towards the moving agents, and analysed its correlations with accuracy and RT differential scores.

**Results**

Paired-sample t-test revealed no significant difference between participants’ likability ratings on the moving and static agents. Also, neither participants’ likability scores nor their TIPI scores can predict the accuracy or RT difference between the moving and static agent’s conditions (all Pearson coefficients are smaller than 0.31, and none of them is significant).

**S6. The gender effect in Experiment 3**

Since we only recruited female participants in Experiment 1 & 2, although we didn’t consider the gender of the participants would confound the result, we decided to balance this factor thus we have both female and male participants in Experiment 3 & 4. We thus analysed our data to explore any significant gender effects. Results showed no significant main effect of gender both on accuracy and reaction time (for accuracy: *F* (1, 35) = 0.615, *p* = 0.438; for RT: *F* (1, 35) = 0.515, *p* = 0.474), nor did participants’ gender interact with the group effect (for accuracy: *F* (1, 34) = 0.192, *p* = 0.664; for RT: *F* (1, 35) = 0.852, *p* = 0.362 ).

**References**

Doosje, B., & Spears, R. (1995). Perceived Intragroup Variability as a Function of Group Status and lcentification. *Journal of experimental social psychology*, *31*, 410-436.

Gosling, S. D., Rentfrow, P. J., & Swann Jr, W. B. (2003). A very brief measure of the Big-Five personality domains. *Journal of Research in personality*, *37*(6), 504-528.

Hertel, G., & Kerr, N. L. (2001). Priming in-group favoritism: The impact of normative scripts in the minimal group paradigm. *Journal of Experimental Social Psychology*, *37*(4), 316-324.

McAuley, E., Duncan, T., & Tammen, V. V. (1989). Psychometric properties of the Intrinsic Motivation Inventory in a competitive sport setting: A confirmatory factor analysis. *Research quarterly for exercise and sport*, *60*(1), 48-58.

1. Here R means this item is reverse-scored. [↑](#footnote-ref-1)
